# Supplementary material for: Atmospheric Ammonia Affects Myofiber Development and Lipid Metabolism in Growing Pig Muscle
Source: Animals (Basel). 2019 Dec 18;10(1):2. doi: 10.3390/ani10010002 (PMC7022806; doi:10.3390/ani10010002)
Supplement: Supplementary file 1 [file animals-10-00002-s001.pdf]

**Table S1.** Composition and nutrient levels of the basal diet (air-dry basis).

| Item | |
|---|---|
| Ingredients, % | |
| Corn | 65.00 |
| Wheat bran | 5.00 |
| Soybean meal | 26.00 |
| Vitamin and mineral premix | 4.00[a] |
| Total | 100 |
| Nutrient level | |
| Digestible energy, Kcal/kg | 3150.00 |
| Crude protein, % | 18.50 |
| Lysine, % | 1.00 |
| Threonine, % | 0.74 |
| Calcium, % | 0.75 |
| Total phosphorus, % | 0.65 |
| Available phosphorus, % | 0.40 |

[a]The premix provided the following per kg of the diet: 100000 IU vitamin A, 50000 IU vitamin D3, 400 mg vitamin E, 30 mg vitamin K3, 45 mg vitamin B1, 100 mg vitamin B2, 70 mg vitamin B6, 0.35 mg vitamin B12, 550 mg nicotinic acid, 450 mg pantothenic acid, 18 mg folic acid, 5 mg biotin, 15 g choline chloride, 1 g Fe, 0.2 g Cu, 1 g Zn, 0.5 g Mn, 3.5 mg I, 2.5 mg Se, 10% Ca, 2.5% total phosphorus, 10% water, 3% Lysine.

**Table S2**. Primer sequences information.

| Genes | Accession | Primer sequences (5’-3’) | Produce size, bp |
|---|---|---|---|
| *GAPDH* | NM_001206359 | Forward: CGTGTCGGTTGTGGATCTGA<br>Reverse: TGACGAAGTGGTCGTTGAGG | 209 |
| *MyHC I* | AB053226 | Forward: AAGGGCTTGAACGAGGAGTAGA<br>Reverse: TTATTCTGCTTCCTCCAAAGGG | 115 |
| *MyHC IIa* | AB025260 | Forward: GCTGAGCGAGCTGAAATCC<br>Reverse: ACTGAGACACCAGAGCTTCT | 137 |
| *MyHC IIx* | AB025262 | Forward: AGAAGATCAACTGAGTGAACT<br>Reverse: AGAGCTGAGAAACTAACGTG | 149 |
| *MyHC IIb* | AB025261 | Forward: ATGAAGAGGAACCACATTA<br>Reverse: TTATTGCCTCAGTAGCTTG | 166 |
| *MSTN* | NM_214435 | Forward: CTCCACTCCGGGAACTGATT<br>Reverse: GAAGATCAGACTCTGTAGGCAT | 129 |
| *Smad2* | NM_001256148.1 | Forward: TGTCGTCCATCTTGCCATTCACTC<br>Reverse: GCTCTCCTCCACCTGCTCCTC | 96 |
| *FoxO1* | NM_214014.3 | Forward: TGTCCTACGCCGACCTCATCAC<br>Reverse: GCACGCTCTTGACCATCCACTC | 96 |
| *MyoG* | NM_001012406.1 | Forward: GGAGAAGCGCAGGCTCAAGAAG<br>Reverse: GCAGGCACTCGATGTACTGGATG | 131 |
| *NR1D2* | XM_021071424.1 | Forward: TGAGATGCAGAGTGCGATGAAGAC<br>Reverse: CTGGACTGGTAAGGCTGTCTGTTC | 106 |
| *IL1RAP* | JF735997.1 | Forward: CGCCTCCTGGTTGTTCTAAGTCC<br>Reverse: GACCGTGAGCACCGTCTTAGC | 183 |
| *IRS1* | NM_001244489.1 | Forward: AGCACAAGCCAGTCCTCTCCTAC<br>Reverse: | 169 |

| | | | |
|---|---|---|---|
| | | CTGCTGGTGGATGACGACGAATC | |
| *FASN* | AY952929.1 | Forward: TGCTCCTCTGTCGCCGTCTATAAG Reverse: TTCCAGGTCAGCCACAGGTAGC | 125 |
| *FOMX1* | XM_021092454.1 | Forward: CCAGTGCCAACCGCTACTTGAC Reverse: GGCAGCAGTGGCTTCATCTTCC | 181 |
| *CCNB3* | XM_013990898.2 | Forward: TGTCCACCACCACTGCTCCTG Reverse: TGCTCCTCCTGTGATCTCTTGCC | 157 |
| *IL18* | NM_213997.1 | Forward: CAGGGACATCAAGCCGTGTT Reverse: TGCCAGACCTCTAGTGAGGC | 114 |
| *SCD* | AY487829 | Forward: TGGTGATGTTCCAGAGGAGGTA Reverse: ATGGCGTAACGAAGAAAGGTG | 139 |

10
